# Excessive annual BMI increase after chemotherapy among young survivors of testicular cancer

**DOI:** 10.1038/sj.bjc.6600714

**Published:** 2003-01-28

**Authors:** C Nord, S D Fosså, T Egeland

**Affiliations:** ^1^Department of Medical Oncology and Radiotherapy, University Hospital, The Norwegian Radium Hospital (NRH), Oslo, Norway; ^2^Section of Medical Statistics, University of Oslo, Oslo, Norway

**Keywords:** testicular cancer, treatment, BMI

## Abstract

Increased body mass index (BMI) is claimed to be a complication among survivors of testicular cancer (TCSs), especially after receiving cisplatin-based chemotherapy. This study compares changes in BMI (kg m^−2^) in TCSs with those observed in age-matched men from the population (controls). Associations between treatment, age and potential BMI changes were sought. In 1999, a survey was performed at the NRH of 444 unilaterally orchiectomised TCSs treated from 1980 to 1990. BMI at survey was recorded in each TCS. Information on principal treatment (surgery only: SURG; radiotherapy only: RAD; chemotherapy ± surgery or radiotherapy: CHEM±) and pretreatment BMI was retrieved from the medical records. The age-matched controls had BMI measurements from population surveys from 1985 and 1996. The annual BMI increase was calculated based on the difference in the two BMI measurements divided by observation time. TCSs displayed a lower pretreatment mean BMI than the controls, whereas no difference was found post-treatment. However, the annual BMI increase in TCSs exceeded that of the controls (0.19 *vs* 0.15, *P*=1.4×10^−7^). The SURG and CHEM± groups showed the greatest annual BMI increase. The multiple regression analysis showed that young TC patients who received chemotherapy displayed an excessive annual BMI increase. Oncologists and young TCSs should be aware of the risk of excessive BMI increase, in particular, after the use of chemotherapy.

The mortality rates because of cancer have decreased in general, and the prevalence of patients with a previous diagnosis of cancer is increasing in society. This means that in future more cured cancer patients will contact their family doctors and specialists because of health problems associated with their history of malignancy. In the last few years, oncologists have thus focused on possible health risks among survivors of cancer, related to the malignancy itself or its treatment.

Testicular cancer (TC) is the most frequent malignancy in men aged 20–40 years, with an incidence rate approaching 9 per 100 000 in Norway (1995–1997, Norwegian Cancer Register, personal communication). After the introduction of cisplatin to the treatment of TC, about 90% of these patients are cured. The prevalence of TC survivors (TCSs) is consequently increasing. For many years oncologists have claimed that TCSs have a similar life expectancy as the age-matched males from the general population. Recent publications (Berger *et al*. 1996; Bokemeyer *et al*. 1996; Talvensaari *et al*. 1996; Gietema *et al*. 1992,^2001^; Petersen *et al*. 1999; Akre *et al*. 2000; Meinardi *et al*. 2000a, 2000b) have, however, described long-term health problems among TCSs. Metabolic and vascular problems have thus been claimed to be associated with the treatment of TC. In particular, patients treated with cisplatin-based chemotherapy are known to have a higher cardiovascular morbidity. It is, however, not clear if the cardiovascular problems seen in TCSs are associated with chemotherapy itself or are a consequence of the metabolic changes (Syndrome X) or are related to both.

In the last decade, an excessive weight increase has been observed in the general population (Tverdal, 2001) and has become a major health problem, as this condition is a significant risk factor for a large number of diseases. In adult individuals, weight gain is reflected by an increase of body mass index (BMI), which represents the principal parameter for WHO's definition of obesity and overweight. Not only do obese patients develop somatic diseases such as Syndrome X, cardiovascular diseases, typeII diabetes mellitus, certain types of cancer and osteoarthritis, but they also suffer from mental distress to a high extent (WHO, 1997). It is therefore of great importance to avoid overweight in general, and to identify those long-term cancer survivors who display an increased risk of developing overweight. For oncologists treating TC, it is therefore of great interest to identify those parameters and in particular those therapeutic modalities that are related to the risk of excessive post-treatment increase of BMI.

Our group has recently performed a long-term follow-up survey on TCSs (Fosså and Dahl, 2002). The obtained data on pre- and post-treatment BMI in TCSs enabled the analysis of the following research questions:

(1) Is there an abnormal BMI increase among long-term TCSs compared to a control group of age-matched men from the normal population?
(2) To what extent is a potential BMI increase related to treatment?

## Patients and Methods

### Eligibility

From 1998 to 2000 surviving Norwegian TC patients, aged 18–75 years and treated between 1980 and 1994, were invited to participate in a national multicentre follow-up survey, which aimed to assess long-term sequelae after treatment of TC (Fosså and Dahl, 2002).

The present paper comprises a subgroup of patients treated for unilateral TC from 1980 to 1990 (T-1) at the Department of Medical Oncology and Radiotherapy at the Norwegian Radium Hospital (NRH), Oslo. Of the invited patients, 81% participated in this study. Patients with bilateral TC or extragonadal germ cell malignancy were excluded, as were those with previous unilateral orchiectomy because of a benign condition and patients who, after their diagnosis of TC, had been treated for a second malignancy (Table 1

**Table 1:** Demographics on controls (HUNT I and II) and all TCSs

	**HUNT**	**All TCSs**	***P***
Age at T-1 (years)	41±12	33±10	<0.000
Age at T-2 (years)	52±12	47±11	<0.000
Marital status; % married at T-2	52	67	<0.000
Education; high school or university (%) at T-2	29	50	<0.000
Observation time (years)	11±1	14±3	<0.000

and Table 2

**Table 2:** Demographics on TCSs according to treatment allocation

	**SURG (92)**	**RAD (170)**	**CHEM±(182)**	***P***
Age at T-1 (years)	30±9	38±10	30±9	<0.00
Age at T-2 (years)	44±9	52±10	44±10	<0.00
Marital status; % married at T-2	67	67	65	0.92
Education; high school or university (%) at T-2	44	50	49	0.45
Observation time (years)	14±3	14±3	14±3	0.55
Nonseminomas	91	2	150	–
Seminomas	1	168	32	–
Stage				
I	87	162	56	–
II	5	7	65	–
III		1	10	–
IV			51	–

).

### Treatment principles

Since 1980, cisplatin-based chemotherapy, retroperitoneal lymph node dissection (RPLND) and infradiaphragmatic radiotherapy have represented the principal postorchiectomy treatment modalities for TC patients at NRH. Patients with early stages of seminoma were in the 1990s treated with infradiaphragmatic radiotherapy, whereas those with advanced stages were treated with chemotherapy, occasionally followed by consolidation radiotherapy. Up to 1987, patients with early stages of nonseminoma underwent primary RPLND, followed by adjuvant chemotherapy in case of metastases. From 1987 onwards diagnostic RPLND was omitted as a routine in nonmetastatic cases, and the surveillance policy or adjuvant chemotherapy was introduced. Metastatic cases with nonseminomatous disease received cisplatin-based chemotherapy, followed by resection of residual tumour manifestations (mostly RPLND). Relapsing patients were treated with additional available chemotherapy. Based on these treatment principles, the TCSs were allocated to three groups: (1) surgery only (SURG); (2) radiotherapy only (RAD) and (3) chemotherapy with or without surgery or radiotherapy (CHEM±) (Table 2).

### Examination at long-term follow-up survey (T-2)

At T-2 (‘post-treatment’) the TCSs went through a physical examination including measurements of height in centimetres and weight in kilogrammes. Figures of weight and height before treatment (‘pretreatment’) were retrieved from the patients' medical records together with the patient's age at the time of treatment (T-1) of TC. The following age classes were defined based on pretreatment age (AGE-1): (1) <29 years; (2) 30–39 years; (3) 40–49 years; (4) >50 years.

### Reference group

The reference group consisted of age-matched men from the HUNT studies (controls). The HUNT studies are health surveys performed in the general population in Nord-Trøndelag, a county in Mid-Norway. The HUNT studies are generally regarded as a survey of ‘Mini-Norway’. The purpose of these surveys was to investigate the average health status in the adult population (age > 20 years) of Nord-Trøndelag. The HUNT studies are composed of two parts: the first part was conducted in 1984–1985 (HUNT I, T-1) and the second part in 1995–1997 (HUNT II, T-2). The survey methods and participation rates are published elsewhere (Midthjell *et al*. 1990,^1999^).

To be eligible as a control person, the man's weight and height should have been recorded at HUNT I and HUNT II. Also, the control person had to be younger than 75 years at T-2. Approximately 3000 control persons did not fulfil these criteria and were therefore excluded. For the purpose of the present study, four age classes were established for the participants in HUNT based on their age at HUNT I (AGE-1: (1) <29 years; (2) 30–39 years; (3) 40–49 years; (4) >50 years; Table 1).

### BMI

For each individual among controls and TCSs, BMI was calculated as weight in kilogrammes divided by the square of height in metres (kg m^−2^) for TCSs and controls. Each TCS and each HUNT participant thus had two recordings of BMI (pre- and post-treatment in TCSs; at HUNT I and HUNT II for controls): BMI-1 and BMI-2, respectively. The values of BMI were categorised according to the recommendations from WHO (1997) regarding the classification of BMI in adults:

Underweight
BMI<18.5
Normal range
BMI 18.5–24.9
Overweight
(without further consideration to defined subgroups) BMI ⩾25.0

Annual BMI change was calculated as the difference between the two BMI measurements divided by observation time in years ((BMI-2 − BMI-1) years^−1^).

## Statistical Methods

The statistical analysis was performed using the package of SPSS 9.0. Descriptive data are presented as mean and standard deviation (s.d.), as well as mean and confidence interval (CI). *χ*^2^ test was used to analyse categorical data. Analysis of variance was used for continuous data and *post hoc* tests were performed with Bonferroni corrections. Linear regression analysis was used to evaluate the impact of independent variables on annual BMI change. All *P*-values were two-tailed. To account for multiple comparisons, a value less than *0.01* was considered as statistically significant.

## Ethics

The study was approved by the institutional and regional ethical committees.

## Results

### TCSs/controls

A total of 444 TCSs and 19 224 controls fulfilled the eligibility criteria for the study (Table 1 and Table 2). The RAD group almost exclusively consisted of patients with seminomas (99%) except for two patients. The SURG group included patients with nonseminomatous disease except for one patient. A total of 82% of the patients from the CHEM± group had a nonseminomatous tumour.

The mean observation time among the TCSs exceeded that of the controls by about 3 years (*P*=1.4×10^−15^). At the time of diagnosis, the TCSs were on average 8 years younger than their controls at HUNT I (*P*=1.1×10^−21^). Of the TCSs, 43% (201) were <29 years at the time of the diagnosis in comparison with 19% (3992) of men aged <29 years in HUNT I. Statistically significant differences of marital status and education levels were found between TCSs and controls.

### BMI-1

**Figure 1:**
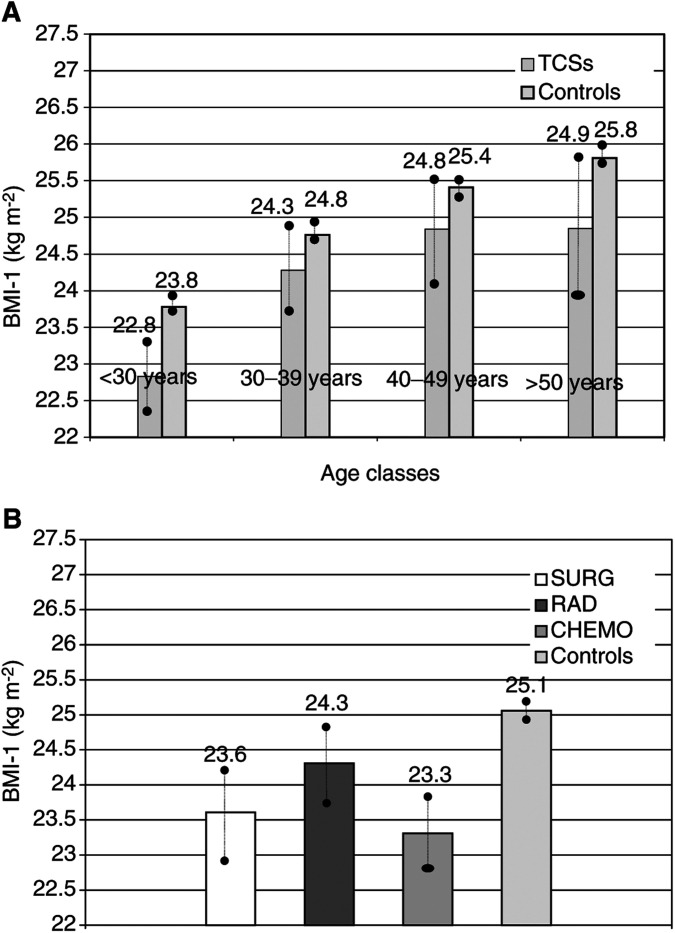
(**A**) Comparison of BMI-1 according to age classes (TCSs and controls: *P*=1.0×10^−15^; •–• 95% CI for mean); (**B**) BMI-1 according to treatment groups (SURG, RAD and CHEM±) and Controls (*P*<0.01; •–• 95% CI for mean).

For all TCSs the mean BMI-1 was significantly below that of all controls (23.8 *vs* 25.1 kg m^−2^; *P*=1.1×10^−15^). For both TSCs and controls, BMI-1 increased with age at T-1, and significant differences of BMI-1 were found between each of the four age classes considering TCSs and controls separately (Figure 1A

). When only comparing BMI values of the youngest age class of TCSs, BMI-1 was significantly below that of the comparable class of the control (*P=*0.00029).

Patients from each of the three treatment groups had a significantly lower BMI-1 than the controls (*P*<0.01; Figure 1B). Patients from the RAD group had the highest mean BMI-1 (*P*=0.0072) and the lowest mean BMI-1 value was observed in the CHEM± group (*P*=5.3×10^−15^).

### BMI-2

**Figure 2:**
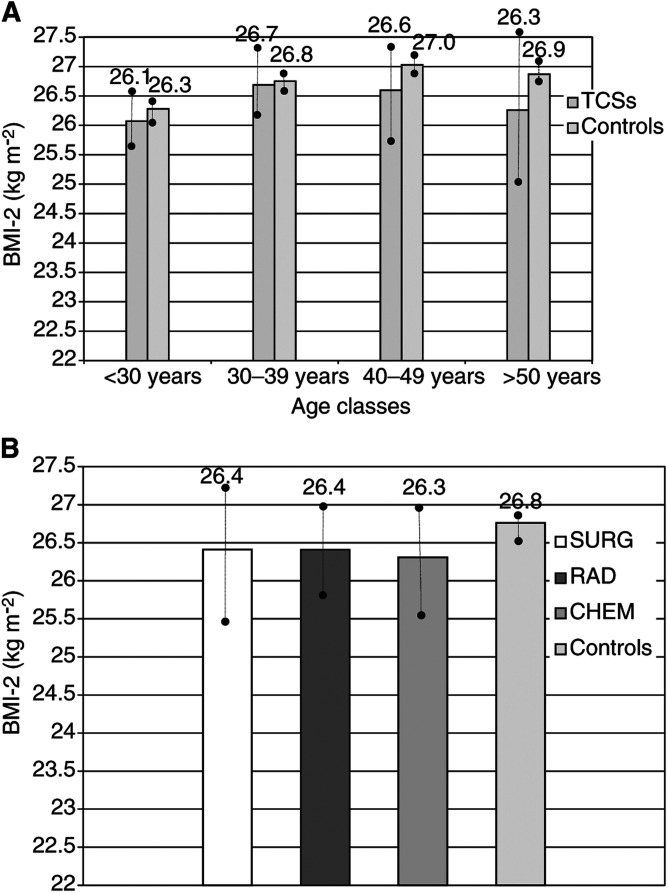
(**A**) Comparison of BMI-2 according to age classes (TCSs and controls: *P*=0.043; •–• 95% CI for mean). (**B**) BMI-2 according to treatment groups (SURG, RAD, CHEM±) and controls (*P*>0.01; •–• 95% CI for mean).

Considering all TCSs together the mean BMI-2 value was below that of the controls, but did not reach our level of statistical significance set at 0.01 (26.4 *vs* 26.7 kg m^−2^; *P*=0.043; Figure 2A

). BMI-2 increased with the person's age at T-1, and significant differences of BMI-2 were found between men from the youngest and the oldest age classes, independent of allocation to patient or control group.

Comparing BMI-2 between TCSs and controls, no significant differences were detectable within any of the four age classes. Even in the youngest age groups of TCSs, BMI-2 was similar to that observed in the controls.

Neither were significant differences of BMI-2 observed between each of the three treatment groups and the controls (*P>*0.01; Figure 2B).

### Annual BMI change

**Figure 3:**
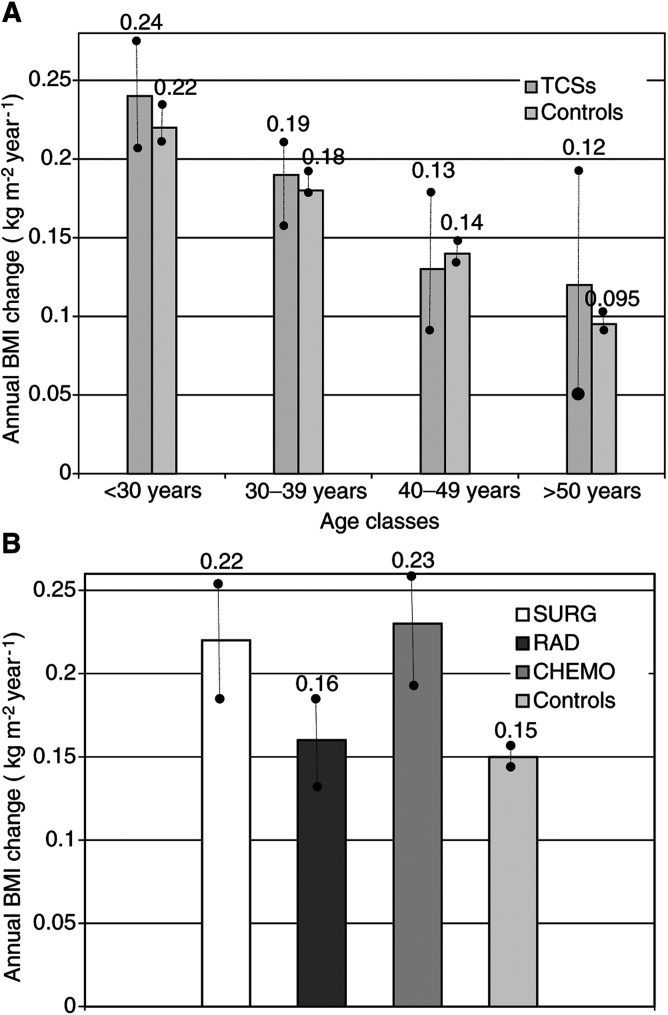
(**A**) Annual BMI increase according to age classes (TCSs and controls: *P*=1.4×10^−7^; •–• 95% CI for mean). (**B**) Annual BMI increase according to treatment groups (SURG, RAD, CHEM±) and controls (*P*<0.01; •–• 95% CI for mean).

In general, the mean annual BMI change was higher among the TCSs than the controls (0.19 *vs* 0.15; *P*=1.4×10^−7^). Age at T-1 was associated with BMI increase: significant differences were observed between the age classes (*P*<0.01), displaying a lower annual BMI increase in the older men than in the younger men. The highest annual BMI changes were found in the two youngest age classes, without statistically significant differences between controls and TCSs (Figure 3A

).

Comparing the three treatment groups to the controls, the SURG group and the CHEM± group displayed the highest value of annual BMI change (*P*=0.00073 resp. *P=*1.5×10^−9^; Figure 3B).

At T-1, 30% of the TCSs and 47% of the controls were classified as overweight (BMI>25 kg m^−2^). At T-2, the comparable percentages had increased to 63% (TCSs) and 71% of the controls (data not shown).

### Annual BMI change, AGE-1 and treatment

AGE-1 as continuous variable and group allocation (SURG *vs* RAD *vs* CHEM± *vs* controls) were entered as independent predictors into the linear regression analysis with annual BMI change as dependent variable, pooling together TCSs and controls. AGE-1 (*P*=1.7×10^−23^) and belonging to CHEM± (*P*=0.013) remained independent predictors of annual BMI change. This model explained only 8% (*R*^2^) of the annual BMI change.

## Discussion

This study shows that TCSs, approximately 14 years after their treatment, displays an abnormal BMI increase compared to the normal population. A high BMI increase is significantly associated with the young age of a TC patient at diagnosis, with chemotherapy as an additional independent parameter. Furthermore, our study confirms previous observations of comparatively low mean BMI in TC patients, in particular prior to treatment, but also >5 years after treatment.

BMI-1 in TCSs was significantly below the average BMI-1 in the controls. Especially, the younger TC patients had a lower BMI-1 than the controls. This surprising observation was also indicated in previous studies from Denmark and Norway (Davies *et al*. 1990; Akre *et al*. 2000). Both studies showed relatively low BMI recordings years before the patients' diagnosis. The reasons for these observations remain unclear. Clinical experience indicates that a poor nutritional status can be excluded as an explanation to TC patients' comparably low BMI at the time of diagnosis. This is supported by a detailed analysis of our BMI-1 and pretreatment weight recordings: no significant differences were found between the four stages of testicular cancer (*P*=0.057 and 0.078, resp.). Subclinical variations of the serum androgens associated both with BMI values and the aetiology of TC may explain some of the observed differences, in particular in young individuals. Relatively high serum testosterone levels, especially during puberty, have been suggested to contribute to the development of TC (Rajpert-De Meyts *et al*. 1993). Moss *et al*.(1986) as well as the United Kingdom Testicular Cancer Study Group (1994) found an association beween young age at puberty and the risk of developing TC, especially nonseminomatous disease. Serum testosterone is inversely associated with BMI (Vermeulen, 1996). Recent observations claim that men with high BMI have a decreased risk of developing TC (Akre *et al*. 2000). On the other hand, another study indicates a decreased risk of developing TC with a more adrenogenic state, such as baldness (Petridou *et al*. 1997). The shown difference in BMI-1 between TCSs and controls could be explained by such hormonal variations.

Epidemiological studies have shown a steady increase of the average BMI in the normal population for the last 20–30 years, and in particular over the last 3–5 years (Jacobsen *et al*. 2001). In general, our TCSs follow this pattern: their post-treatment BMI is above their pre-treatment BMI. Post-treatment BMI values that exceed those of the general population have been described as a long-term problem specific for TCSs, in particular after cisplatin-based chemotherapy (Gietema *et al*. 1992). We could not confirm these observations comparing BMI-2 in all TCSs and controls. On the contrary, BMI-2 values in TCSs were significantly below those of the controls. A possible explanation for these diverse results may be that not all reports have included adequate control groups.

It is extremely difficult to establish an adequate control group measuring BMI in age-matched men from the normal population performed approximately at the same time as in TCSs. Even the present study suffers from some shortcomings in that respect. At T-2, the controls in our study were on average 8 years older than TCSs ( Table 1). Furthermore, BMI-2 values of the controls were obtained in 1995–1997 after an observation time that was 3 years shorter than that on average in TCSs. Increasing age is associated with increasing BMI until at least the 5–6th decade of life (Jacobsen *et al*. 2001), whereas shorter observation time is associated with a lower BMI value. As the effect of these parameters (age, observation period and year to BMI measurements) points in opposite directions, we nevertheless consider our control group as adequate for BMI-2 comparisons between TCSs and controls. Significant differences were also found when comparing marital status and education level between TCSs and controls. A low BMI is associated with marital status as well as high education (Tverdal, 2001). These two factors are over-represented among the TCSs at T-2, which could to some extent explain the lower mean BMI-2 value, although we consider this impact as rather limited.

After their treatment, TCSs do increase their BMI to a higher extent than the controls. In our study, low age was the most important predictive factor for a high BMI increase. In general, BMI tends to rise more during adolescence and early adulthood than during later life (WHO, 1997). Some of the observed differences between TCSs and controls may thus be because of the above-mentioned 8-year age difference of our main cohorts. On the other hand, any clinical or subclinical postorchidectomy hormone changes, if of impact on BMI development, may be more pronounced in the younger than in the older TC patient. Androgen insuffiency is, for example, often associated with overweight, and may after orchidectomy be of particular importance in the youngest age classes of TCSs, when serum testosterone levels usually are high. Patients younger than 30 years are those who have the highest risk of cancer *in situ* in the contralateral testicle or are those who develop a new TC (Hoff Wanderås *et al*. 1997). This indicates that these patients most probably more often than the older ones have histopathological and functional abnormalities in the contralateral testicle, including inadequate Leydig cell function (Bokemeyer *et al*. 1996). Owing to these conditions, these young patients more often than the older ones may suffer from subclinical androgen insufficiency after unilateral orchidectomy.

It has earlier been argued that cisplatin-based chemotherapy in particular leads to an abnormal BMI increase in TCSs possibly related to hormonal changes that in turn lead to post-treatment weight gain (Gietema *et al*. 1992). During recent years, it has been pointed out that cisplatin-based chemotherapy may lead to subclinical Leydig cell function (Vejby Hansen *et al*. 1989). A significant difference concerning body fat has been reported in TC patients who received chemotherapy at an age below 30 years compared to those treated by orchidectomy only (Gietema *et al*. 1992). Our data support these observations to some extent. In the linear regression analysis comprising all men from the control group and all TCSs, young age was associated with a high annual BMI increase. In addition, chemotherapy remained an additional independent factor predicting a high annual BMI increase. Young age is thus a ‘risk factor’ for high annual BMI increase, which furthermore increases with chemotherapy.

Another important difference between the Gietema *et al*.(1992) study and ours was that the average observation period in the former study was only 3 years as compared to 14 years in our study. Any hormonal or metabolic changes leading to post-treatment BMI increase may be more pronounced shortly after chemotherapy than later on in life (Gietema *et al*. 1992; Berger *et al*. 1996).

Except for post-treatment hormone changes, one can only speculate about other reasons for the high BMI increase in TCSs, based on observations in other cancer survivors. It is known that women with breast cancer, receiving adjuvant chemotherapy, might develop excessive weight gain (Goodwin *et al*. 1988; Jernström *et al*. 1999). Overeating and less physical activity owing to mental distress have been discussed together with hormonal changes. A more recent theory is that chemotherapy would induce hyperphagia (Demark-Wahnefried *et al*. 1993). All these parameters may explain the observed BMI increase in TCSs (Gietema *et al*. 1992), especially in the younger ones, who have highest levels of anxiety and fatigue (Fosså *et al*. 2002). Adolescence and early adulthood are known to be critical periods for the development of obesity (WHO, 1997).

According to WHO (1997), abnormal BMI increase in a person as such is a serious health problem, not only the final result of such development: overweight and obesity. In particular, any weight gain at an early age is known to be a major health risk and could contribute to the development of Syndrome X.

In conclusion, although post-treatment BMI values in long-term TCSs do not exceed that of the normal age-matched male population, the annual BMI increase in TCSs tends to exceed that of the control group. Patients treated below the age of 30 years and, in particular, after the application of chemotherapy, are at a high risk of abnormal BMI increase. Physicians treating TC should be aware of this health risk and advise their patients accordingly to avoid subsequent somatic and psychological disabilities related to overweight and obesity.
